# Prediction model of hepatocellular carcinoma risk in Asian patients with chronic hepatitis B treated with entecavir

**DOI:** 10.18632/oncotarget.21369

**Published:** 2017-09-28

**Authors:** Chien-Hung Chen, Chuan-Mo Lee, Hsueh-Chou Lai, Tsung-Hui Hu, Wen-Pang Su, Sheng-Nan Lu, Chia-Hsin Lin, Chao-Hung Hung, Jing-Houng Wang, Mei-Hsuan Lee, Cheng-Yuan Peng

**Affiliations:** ^1^ Division of Hepatogastroenterology, Department of Internal Medicine, Kaohsiung Chang Gung Memorial Hospital and Chang Gung University College of Medicine, Kaohsiung, Taiwan; ^2^ Division of Hepatogastroenterology, Department of Internal Medicine, China Medical University Hospital, Taichung, Taiwan; ^3^ Institute of Clinical Medicine, National Yang-Ming University, Taipei, Taiwan; ^4^ School of Medicine, China Medical University, Taichung, Taiwan

**Keywords:** nucleos(t)ide analog, hepatocellular carcinoma, risk score, platelet, alpha-fetoprotein

## Abstract

**Background:**

Until now, no risk score could predict hepatocellular carcinoma (HCC) in nucleos(t)ide analog (NA)-treated Asian patients.

**Methods:**

We enrolled 1325 NA-naïve chronic hepatitis B patients with entecavir monotherapy for >12 months, with 883 and 442 patients randomly assigned to the development and validation groups, respectively, in the risk model.

**Results:**

The cumulative probabilities of HCC were 2.4%, 4.1%, and 9.9% after 2, 3, and 5 years of treatment, respectively. In the development group, age, platelet counts, and alpha-fetoprotein levels after 12 months of treatment were the independent predictors of HCC. We converted the Cox proportional hazards regression coefficients for these predictors into risk scores and developed the APA-B model, with the total risk scores ranging from 0 to 15. The risk scores accurately categorized patients with low (0–5), medium (6–9), and high (10–15) risks in the validation group (*P* <0.001). The areas under the receiver operating characteristic curve for predicting HCC risk after 2, 3, and 5 years were 0.877, 0.842, and 0.827, respectively, in the development group and 0.939, 0.892, and 0.862, respectively, in the validation group.

**Conclusion:**

The proposed HCC risk prediction model exhibited excellent predictive accuracy in NA-naïve Asian patients receiving entecavir therapy.

## INTRODUCTION

Hepatitis B virus (HBV) infection is a major health concern, and hepatocellular carcinoma (HCC) remains a major cause of morbidity and mortality worldwide [1, 2]. Entecavir (ETV) is one of the most widely used potent oral nucleos(t)ide analogs (NAs) for treating HBV [3]. Long-term ETV therapy results in histological improvements, leading to the regression of fibrosis and cirrhosis [4]. Long-term ETV therapy has been reported to reduce the incidence of HCC in Asian patients with HBV-related cirrhosis [5, 6]. However, HCC may still develop in patients with chronic hepatitis B (CHB), particularly in those with preexisting cirrhosis [7, 8]. Until now, three risk scores, namely GAG-HCC, CU-HCC, and REACH-B, have been developed for predicting HBV-related HCC in untreated Asian patients with CHB [9–11]. However, recent studies have reported controversial results regarding the performance of these risk scores in patients receiving ETV therapy [12–14]. Studies on HCC development in patients with CHB have enrolled both NA-naïve and -experienced patients in addition to patients receiving different NAs, which might have influenced HCC development because of the varying rates of resistance mutation and virological suppression [12–14]. Therefore, a large cohort study conducted in a real-life setting that involves only NA-naïve patients receiving a single session of NA therapy is required for developing a new risk score for predicting HCC in patients receiving long-term NA therapy.

This study investigated the incidence and predictors of HCC and developed a new, readily applicable HCC risk score for NA-naïve patients receiving long-term ETV therapy.

## RESULTS

### Clinical characteristics of the patients

Of the 1325 NA-naïve patients, 963 were men and 362 were women (median age, 50 ± 17 years). The median treatment duration was 49.1 (12–130.6) months, which equaled the observation period for HCC occurrence because this study aimed to investigate the incidence of HCC during therapy. Table 1 shows the baseline clinical characteristics of the patients. The cumulative rates of the virological response (VR) of the 475 hepatitis B e antigen (HBeAg)-positive patients after 1, 3, and 5 years were 74.5%, 94.4%, and 97.4%, respectively. The cumulative rates of HBeAg loss after 1, 3, and 5 years were 21.4%, 45.8%, and 61.9%, respectively. Furthermore, the cumulative rates of the VR of the 850 HBeAg-negative patients after 1, 3, and 5 years were 94.4%, 99.2%, and 99.5%, respectively. Of all patients, seven demonstrated ETV resistance; the 7-year cumulative incidence of ETV resistance was 0.84%. All received rescue therapy and achieved VR subsequently (1 with ETV plus adefovir, 3 with ETV plus adefovir then switch to tenofovir, and 3 with tenofovir). A total of 105 patients developed HCC during therapy. None of the patients with ETV resistance developed HCC. The cumulative rates of HCC at 2, 3, 5, and 7 years were 2.4%, 4.1%, 9.9%, and 13%, respectively.

**Table 1:** Baseline characteristics of all patients

Variables Median ± IQR or *n* (%)	All patients *n* = 1325
Age (year)	50 ± 17
Sex, male	963 (72.7)
HBeAg-positive status	475 (35.8)
Cirrhosis status	481 (36.3)
Diabetes mellitus, yes	158 (11.9)
Family history of HCC, yes	257 (19.4)
Albumin, g/dL	4.1 ± 0.6
AST, U/L	72 ± 115
ALT, U/L	106 ± 207
AAR	0.71 ± 0.39
Total bilirubin, mg/dL	1.00 ± 0.78
INR	1.08 ± 0.15
Platelet, ×10^3^/μL	162 ± 76
AFP, ng/mL	6.01 ± 10.31
Genotype	
B	859 (64.8)
C	466 (35.2)
HBV DNA, log_10_ IU/mL	5.96 ± 2.38
HBsAg, log_10_ IU/mL	3.29 ± 0.9

Abbreviations: AFP, alpha-fetoprotein; AST, aspartate aminotransferase; ALT, alanine aminotransferase; AAR, AST/ALT ratio; HBV, hepatitis B virus; HCC, hepatocellular carcinoma; HBeAg, hepatitis B e antigen; HBsAg, hepatitis B surface antigen; INR, international normalized ratio; IQR, interquartile range.

### HCC risk prediction model of the development group

The clinical characteristics were comparable between the development and validation groups (Supplementary Table 1). The cumulative incidences of HCC at 2, 3, 5, and 7 years were 2.2%, 3.4%, 10.3%, and 13.7%, respectively, in the development group (*n* = 70). In the validation group (*n* = 35), the cumulative incidences of HCC were 2.8%, 5.4%, 9.2%, and 11.9% at 2, 3, 5, and 7 years, respectively (*P* = 0.920; Supplementary Figure 2).

Univariate and multivariate analyses of factors associated with HCC in the development group revealed two alternative models, which considered age, platelet counts, and alpha-fetoprotein (AFP) levels at baseline and 12 months as the independent predictors of HCC (Table 2 and Supplementary Table 2). To identify the most favorable model for predicting the 5-year HCC risk in the development group, we compared the areas under the receiver operating characteristic curve (AUROCs) of age, platelet counts, and AFP levels for predicting HCC at baseline and 12 months (Supplementary Table 3). Both platelet counts and AFP levels showed higher AUROCs at 12 months than at baseline. Furthermore, the AUROCs of the full logistic regression model comprising all three predictors of HCC at baseline and 12 months were 0.807 and 0.863, respectively (*P* < 0.05; Supplementary Table 3). Thus, we constructed the HCC risk prediction model on the basis of age, platelet count, and AFP level at 12 months of treatment.

**Table 2:** Univariate and multivariate analyses of factors associated with hepatocellular carcinoma in the development group (*n* = 883)

	Univariate analysis		Multivariate analysis	
Variables	Hazard ratio (95% CI)	*P* value	Hazard ratio (95% CI)	*P* value
Age (year)	1.058 (1.036–1.080)	< 0.001		
Age at 12 months (year)	1.058 (1.036–1.080)	< 0.001	1.036 (1.014–1.059)	0.001
Sex, male *vs* female	1.015 (0.593–1.737)	0.958		
HBV genotype, C *vs* B	1.472 (0.919–2.357)	0.105		
HBeAg, yes or no	0.704 (0.412–1.205)	0.201		
HBV DNA, per log_10_ IU/mL	0.827 (0.704–0.971)	0.021		
Diabetes mellitus, yes *vs* no	2.640 (1.510–4.615)	0.001		
Family history of HCC, yes *vs* no	1.479 (0.846–2.586)	0.170		
Platelet, per 10^3^/μL	0.984 (0.980–0.989)	< 0.001		
Platelet at 12 months, per 10^3^/μL	0.977 (0.972–0.982)	< 0.001	0.978 (0.973-0.983)	< 0.001
AST, per U/L	0.999 (0.997–1.000)	0.068		
ALT, per U/L	0.998 (0.996–0.999)	0.006		
AAR	2.590 (1.825–3.677)	< 0.0001		
AAR at 12 months	2.180 (1.263–3.518)	0.0043		
Total bilirubin, per mg/dL	0.947 (0.827–1.085)	0.435		
Albumin, per g/L	0.386 (0.260–0.573)	< 0.001		
INR, per ratio	2.088 (1.001–4.355)	0.050		
AFP at baseline, per ng/mL	1.005 (0.997–1.013)	0.190		
AFP at 12 months, per ng/mL	1.008 (1.005–1.011)	< 0.001	1.007 (1.004–1.010)	< 0.001
HBsAg at baseline, per log_10_ IU/mL	0.793 (0.595–1.056)	0.112		
HBsAg at 12 months, per log_10_ IU/mL	0.845 (0.612–1.165)	0.303		
Time to VR, month	0.997 (0.966–1.029)	0.847		
VR at 6 months, yes *vs* no	1.146 (0.664–1.980)	0.624		
VR at 12 months, yes *vs* no	1.861 (0.678–5.106)	0.228		

Abbreviations: AFP, alpha-fetoprotein; AST, aspartate aminotransferase; ALT, alanine aminotransferase; CI, confidence interval; AAR, AST/ALT ratio; HBV, hepatitis B virus; HBeAg, hepatitis B e antigen; HBsAg, hepatitis B surface antigen; HCC, hepatocellular carcinoma; INR, international normalized ratio; VR, virological response.

To include an appropriate number of HCC cases in each stratum, we used the 50th and 75th percentiles of the platelet counts and the 25th and 75th percentiles of the AFP levels of the patients who developed HCC during the therapy as the cutoff values. Thus, we used 100 × and 130 × 10^3^/μL as the cutoff values for platelet counts and 5 and 9 ng/mL as the cutoff values for AFP levels at 12 months of treatment. Approximately 4.2% (28/672), 16.7% (27/162), and 30.6% (15/49) of patients with AFP levels of < 5, 5–9, and > 9 ng/mL, respectively, developed HCC (*P* < 0.001); the median treatment duration was 42.6 ± 21, 38.1 ± 30, and 20.5 ± 30.2 months for these patients. The remaining patients did not exhibit evidence of HCC after the median treatment duration of 47.8 ± 36, 55 ± 36, and 51.5 ± 41.7 months. We computed integer risk scores in the risk prediction model by converting the regression coefficients of the independent predictors at 12 months of treatment (Table 3). We named the developed HCC risk prediction model the APA-B score; the model was constructed on the basis of the independent predictors of age, platelet count, and AFP level at 12 months of treatment. The total risk scores ranged from 0 to 15 in this model. Table 4 lists the predicted HCC risks after 2–5 years of ETV treatment for the total risk score spectrum. The C-statistic of the model was 0.85. Internal validation was performed using the bootstrap method; the resulting C-statistic was 0.85.

**Table 3:** Multivariate Cox regression analyses of on-treatment factors associated with hepatocellular carcinoma in the development group (*n* = 883)

Variables	Hazard ratio (95% CI)	Parameter	*P* value	Risk scores
Age at 12 months, year	1.422 (1.135–1.783)	0.3524	0.002	
<40				0
40–49				1
50–59				2
60–69				3
≥70				4
Platelet at 12 months, 10^3^/μL				
≥130	1.000			0
100–129	2.597 (1.245–5.417)	0.9544	0.011	3
<100	8.892 (4.483–16.327)	2.1851	< 0.001	6
AFP at 12 months, ng/mL				
<5	1.000			0
5–9	2.124 (1.229–3.671)	0.7534	0.007	2
>9	4.900 (2.578–9.316)	1.5893	< 0.001	5

The proportionality assumption of Cox models was examined, and the assumption was not violated.

Abbreviations: AFP, alpha-fetoprotein; CI, confidence interval.

**Table 4:** Total risk score and the predicted 2–5-year risk of hepatocellular carcinoma in the development group (*n* = 883)

Risk scores	Year 2	Year 3	Year 4	Year 5
**0**	0.5%	0.8%	1.8%	2.4%
**1**	0.8%	1.2%	2.5%	3.5%
**2**	1.1%	1.7%	3.6%	4.9%
**3**	1.5%	2.4%	5.1%	6.9%
**4**	2.2%	3.4%	7.1%	9.7%
**5**	3.1%	4.7%	10.0%	13.4%
**6**	4.3%	6.7%	13.9%	18.6%
**7**	6.1%	9.3%	19.1%	25.3%
**8**	8.6%	13.0%	26.1%	34.0%
**9**	12.0%	18.0%	34.9%	44.6%
**10**	16.6%	24.6%	45.7%	56.9%
**11**	22.8%	33.1%	58.1%	69.8%
**12**	30.8%	43.5%	70.9%	81.8%
**13**	40.8%	55.6%	82.8%	91.1%
**14**	52.5%	68.5%	91.8%	96.8%
**15**	65.3%	80.7%	97.1%	99.3%

**Figure 1:**
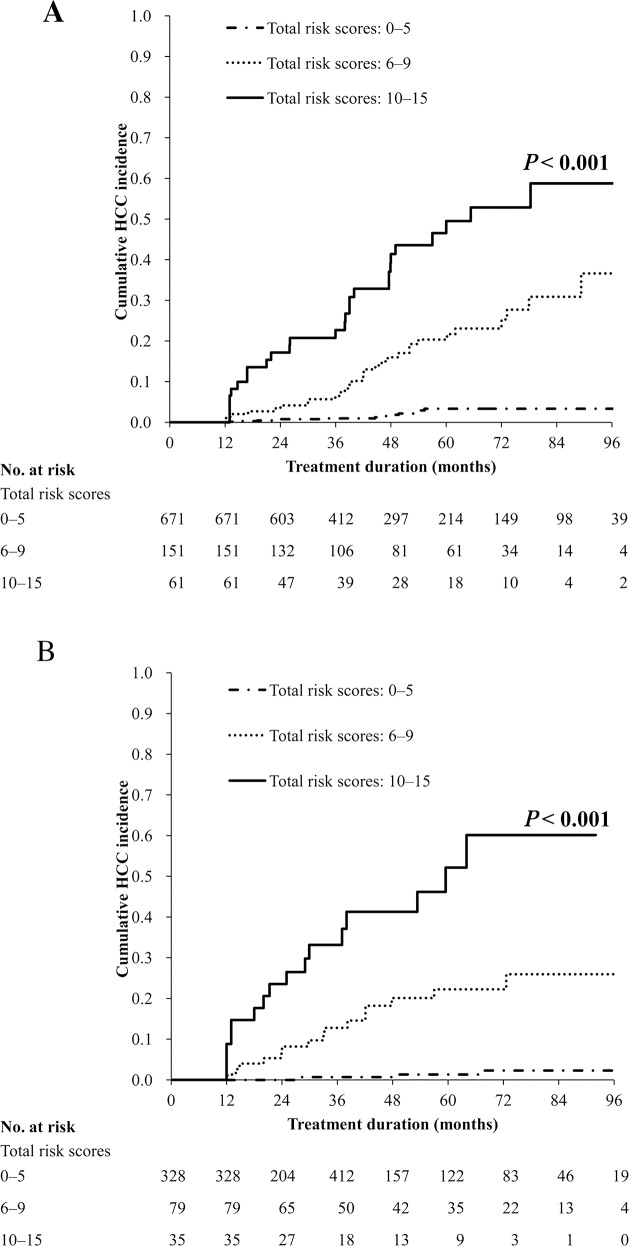
Cumulative HCC incidence rates according to the total risk scores in the **(A)** development and **(B)** validation groups.

To include an appropriate number of HCC cases in each stratum, we used the 25th and 75th percentiles of the risk scores of these HCC cases as the cutoff values; thus, we used 6 and 10 as the cutoff risk scores. On the basis of their total risk scores, patients in the development group were further categorized into the following subgroups: low (0–5); medium (6–9); and high (10–15) risk. The high-risk score group showed a higher cumulative HCC incidence (*P* **<** 0.001; Figure 1A). The model exhibited excellent calibration capability in the development group (all *P* > 0.05 for 2–5 years).

### Validation of the HCC risk prediction model

The AUROCs for predicting the 2-, 3-, and 5-year HCC risks in the development group were 0.877, 0.842, and 0.827, respectively. In the validation group, the AUROCs for predicting the 2-, 3-, and 5-year HCC risks were 0.939, 0.892, and 0.862, respectively (Table 5). The C-statistic of the model was 0.87 for the validation group. On the basis of their risk scores, patients in this group were further categorized and assigned to one of the following subgroups: low (0–5), medium (6–9), and high (10–15) risk. The observed cumulative incidence curves for these subgroups significantly differed (*P* < 0.001; Figure 1B). Furthermore, the model exhibited excellent calibration capability in the validation group (all *P* > 0.05 for 2–5 years). Supplementary Table 4 presents the sensitivity, specificity, positive predictive value, and negative predictive value (NPV) for HCC development within the initial 5 years of ETV therapy in the development and validation groups that were determined using ≥ 6 as the cutoff value of the APA-B score.

**Table 5:** Time-dependent AUROCs for predicting hepatocellular carcinoma by using different risk scores

	APA-B	CU-HCC	REACH-B I	REACH-B II	PAGE-B
Development Group (*n* = 883)	AUROC (95% CI)	AUROC (95% CI)	AUROC (95% CI)	AUROC (95% CI)	AUROC (95% CI)
2 years	0.877 (0.789–0.965)	0.806 (0.731–0.882)	0.640 (0.507–0.774)	0.693 (0.554–0.832)	0.788 (0.707–0.870)
3 years	0.842 (0.771–0.914)	0.767 (0.685–0.850)	0.616 (0.506–0.726)	0.639 (0.526–0.752)	0.742 (0.650–0.834)
4 years	0.857 (0.806–0.907)	0.777 (0.716–0.838)	0.639 (0.553–0.724)	0.645 (0.564–0.727)	0.733 (0.664–0.803)
5 years	0.827 (0.771–0.883)	0.760 (0.698–0.821)	0.620 (0.535–0.705)	0.638 (0.561–0.715)	0.696 (0.620–0.773)
**Validation Group(*n* = 442)**	**AUROC (95% CI)**				
2 years	0.939 (0.902–0.976)				
3 years	0.892 (0.828–0.956)				
4 years	0.883 (0.829–0.938)				
5 years	0.862 (0.795–0.929)				

Abbreviations: AUROC, the area under the receiver operating characteristic curve; CI, confidence interval.

### AUROC and C-statistic comparison of different HCC risk prediction models

The AUROCs for predicting 2-, 3-, and 5-year HCC risks were 0.806, 0.767, and 0.760, respectively, according to the CU-HCC score [10]. According to the REACH-B I score, the AUROCs for predicting 2-, 3-, and 5-year HCC risks were 0.640, 0.616, and 0.620, respectively [11]. Furthermore, the AUROCs for predicting 2-, 3-, and 5-year HCC risks were 0.693, 0.639, and 0.638, respectively, based on the REACH-B II score [15]; the AUROCs were 0.788, 0.742, and 0.696 according to the PAGE-B score (Table 5) [16]. The AUROCs between the APA-B and other models at 2–5 years significantly differed (Supplementary Table 5). In the development group, the C-statistics of the APA-B, CU-HCC, REACH-B I, REACH-B II, and PAGE-B models were 0.85 (95% confidence interval [CI]: 0.81–0.88), 0.74 (95% CI: 0.68–0.80), 0.61 (95% CI: 0.50–0.73), 0.64 (95% CI: 0.56–0.72), and 0.70 (95% CI: 0.61–0.79), respectively. The APA-B model yielded a significantly higher C-statistic than did the other models. The GAG-HCC score could not be determined because the status of the basal core promoter mutation was unavailable in our cohort.

## DISCUSSION

Our study revealed that the cumulative HCC rates for all patients were 2.4%, 4.1%, 9.9%, and 13% after 2, 3, 5, and 7 years of treatment, respectively. Thus, these patients should be regularly monitored for HCC, even when they are receiving ETV therapy [17].

Several scoring systems (i.e., GAG-HCC, CU-HCC, and REACH-B) are available; however, they have been developed by analyzing data from untreated Asian patients [9–11]. Recent studies have reported the suboptimal performance of these models for predicting HCC in NA-treated Caucasian patients [13, 14]. Thus, a new risk score is required for predicting HCC during long-term NA therapy. The present study revealed that old age, lower platelet counts, and higher AFP levels both at baseline and 12 months are independent predictors of HCC. Previous studies have identified age and the severity of liver diseases as the major risk factors for HCC in patients with CHB receiving NA therapy [13, 14, 16, 18, 19]. However, cirrhosis had been mostly diagnosed by clinical and imaging criteria in these studies, which conferred some diagnostic inaccuracy. In addition, several recent studies have identified lower platelet counts as an independent factor associated with an elevated HCC risk during ETV or tenofovir therapy [5, 13, 14, 16]. A study reported an inverse correlation between platelet counts and the hepatic venous pressure gradient in patients with compensated cirrhosis [20]. For patients with compensated cirrhosis, another study identified the hepatic venous pressure gradient as an independent predictor of HCC [21]. Thus, platelet counts most likely reflect the severity of liver fibrosis and can serve as a surrogate marker of HCC risk. To overcome the diagnostic uncertainty of cirrhosis imposed through ultrasonography, we employed two simple noninvasive fibrosis indices, platelet count and aspartate aminotransferase (AST)/alanine aminotransferase (ALT) ratio (AAR), as a marker of liver fibrosis and investigated their predictive roles for HCC [22]. We demonstrated that platelet count was an independent predictor of HCC and that the cutoff values of 100 × and 130 × 10^3^/μL for platelet counts stratified the risk of HCC in the risk prediction model. Furthermore, we observed that platelet count at 12 months of treatment was a significantly stronger predictor than that at baseline. Platelet counts at 12 months of treatment may obviate the confounding influence of necroinflammation on fibrosis measurement and thus more appropriately reflect the actual degree of baseline fibrosis. Alternatively, platelet counts at 12 months of treatment reflect the degree of residual fibrosis with ongoing NA therapy and thus more strongly correlate with future HCC risk. Additional studies are needed to elucidate the underlying mechanisms. AFP has long been used in the surveillance of HCC and evaluation of treatment responses in patients with HCC. Several recent studies have reported that the on-treatment AFP level is a strong predictor of HCC in patients with CHB receiving ETV therapy [23–25]. We observed that AFP levels were more predictive of HCC during ETV therapy at 12 months than at baseline; this is because the elevated AFP levels in patients commencing antiviral therapy were related to the hepatitis activity and were minimized through NA therapy. However, elevated AFP levels at 12 months did not necessarily imply the existence of HCC, considering that only 19.9% (42/211) of the patients with AFP levels of ≥ 5 ng/mL at 12 months in the development group developed HCC. The remaining patients exhibited no evidence of HCC despite a significantly longer follow-up duration (33 ± 30 *vs* 53 ± 36 months, *P* < 0.001). Our findings reveal that AFP cutoff values of 5 and 9 ng/mL at 12 months of treatment were optimal for predicting HCC risk during ETV therapy.

Although studies have reported a significant association between hepatitis B surface antigen (HBsAg) levels and an increased risk of cirrhosis and HCC in untreated patients [15, 26], we observed that HBsAg levels both at baseline and 12 months were not independent predictors of HCC during ETV therapy. Studies have reported that a VR is associated with a lower HCC risk during ETV therapy [6, 27]. However, we observed that a VR after 6 or 12 months of ETV treatment or the time to VR could not predict HCC, which is consistent with recent studies on Caucasian patients [7, 16]. This might be due to the inclusion of NA-experienced patients in previous studies [6, 27]. Our previous study revealed that the VR to ETV therapy was a significantly negative predictor of HCC only in NA-experienced patients and not in NA-naïve patients [8]. The male sex is an established risk factor for HCC in patients with untreated CHB [9, 11]. Recent evidence reported that the androgen–androgen receptor complex can upregulate HBV RNA transcription and enhance HBV DNA replication, which at least partially explains its mechanism of action [28]. Several recent cohort studies on patients receiving ETV therapy have not identified the male sex as a risk factor for HCC [6, 13, 24, 25], which is in accordance with the present finding. A hypothesis posits that NA therapy might inhibit the influence of androgen on HBV replication, consequently minimizing its effect on hepatocarcinogenesis.

We developed and validated the APA-B score at 12 months of treatment for estimating the HCC risk up to a 5-year endpoint for ETV-treated patients with CHB. The on-treatment prediction model exhibited a significantly higher predictive value than that of the baseline model. The APA-B score yields the most favorable AUROCs compared with the extant HCC risk scores [10, 11, 15, 16]. An APA-B score of ≥ 6 had a sensitivity of 78.3% and 90.3% and an NPV of 98.1% and 99.1% for predicting HCC in the development and validation groups, respectively. Thus, presently, the APA-B score is the most favorable model for predicting HCC in NA-naïve Asian patients receiving ETV therapy.

Our study enrolled patients without cirrhosis and those with compensated cirrhosis, reflecting the distribution of patients in clinical practices. Patients with early cirrhosis might have been underdiagnosed because only 25.4% (122/481) of our patients with compensated cirrhosis were histologically diagnosed. However, we adopted platelet count as a noninvasive, readily applicable, and reproducible test to measure the degree of fibrosis in all enrolled patients to overcome the diagnostic uncertainty of cirrhosis imposed through ultrasonography. Although all enrolled patients were advised not to use herbal medicine throughout ETV treatment and claimed to follow the advice, the possible impact of secret herbal medicine use on HCC risk cannot be completely ruled out [29]. Compared with previous studies, this study enrolled a large homogeneous cohort of NA-naïve Asian patients with CHB with the longest timespan for receiving ETV therapy. The risk score model includes three parameters that can be determined with relative ease and reproducibility, thus providing clinicians with a convenient, inexpensive tool for stratifying HCC risk. Risk stratification may facilitate patient counseling and provide the basis for designing different surveillance strategies and implementing the chemoprevention of HCC in future studies. However, the further substantiation of its predictability is warranted for patients of other ethnicities as well as for those receiving long-term tenofovir therapy.

In conclusion, in this cohort study conducted in a real-life setting, we incorporated risk factors to develop an on-treatment risk score model for HCC during ETV therapy. The APA-B score exhibited excellent predictive accuracy and discriminatory capability, which may enable clinicians to identify patients at a high risk of HCC in the antiviral therapy era.

## MATERIALS AND METHODS

### Patients

From January 2007 to August 2012, we retrospectively enrolled 729 and 596 consecutive NA-naïve patients with CHB commencing ETV monotherapy at Kaohsiung Chang Gung Memorial Hospital and China Medical University Hospital, respectively. The inclusion criteria included seropositivity for HBsAg for > 6 months, a viral load of ≥ 2000 IU/mL at the initiation of ETV therapy, and a treatment duration of > 12 months. Patients had been confirmed to be not using herbal medicine at the initiation of ETV therapy and advised not to use herbal medicine throughout treatment course. Patients were excluded if they exhibited any evidence of other forms of liver diseases, decompensated cirrhosis, had HCC at baseline or within the initial 12 months of ETV therapy, or had a history of immunosuppressive therapy (Supplementary Figure 1).

To develop the HCC risk prediction model, we randomly assigned all patients to the model development or validation group in a 2:1 ratio using the RANUNI function in SAS. The baseline and on-treatment host and viral profiles of patients in the development group were used to establish the prediction model, whereas those of patients in the validation group were used to assess its predictive accuracy.

Cirrhosis was diagnosed according to either (1) histology (*n* = 122) or (2) repeated ultrasounds with consistent findings suggestive of cirrhosis in addition to clinical features such as splenomegaly, thrombocytopenia, ascites, or gastroesophageal varices [30]. Decompensated cirrhosis was defined on the basis of a history or current evidence of ascites, variceal hemorrhage, or hepatic encephalopathy. A family history of HCC was retrospectively reviewed and considered valid if at least one parent or sibling of the index patient had been diagnosed. Every 3–6 months, AFP levels were measured with liver ultrasound for HCC surveillance. HCC was diagnosed according to the practice guidelines of the American Association for the Study of Liver Diseases [17]. This study was conducted in accordance with the 1975 Declaration of Helsinki. All patients provided written informed consent, and the study was approved by the Research Ethics Committees of Chang Gung Memorial Hospital and China Medical University Hospital.

### Methods

All patients were followed every 12 weeks throughout the therapy and more frequently if clinically indicated. Follow-up examinations included a clinical assessment and conventional liver biochemical and serological tests. HBV DNA levels were determined at baseline and every 6 months during the therapy or at the time of biochemical breakthrough. Serum HBsAg was quantified at baseline and after 12 months of therapy.

### Definitions

The VR was defined as a serum HBV DNA level of < 50 IU/mL during ETV therapy [8]. Virological breakthrough was defined as an increase in serum HBV DNA levels of ≥ 1 log_10_ IU/mL from nadir.

### Serology

HBeAg and anti-HBe antibodies were detected using commercial assay kits (Abbott, North Chicago, IL, USA). The HBsAg titers were measured using Abbott Architect HBsAg QT assays (dynamic range: 0.05–250 IU/mL). Serum HBV DNA was quantified using the COBAS Amplicor HBV monitor test (lower limit of detection: 50 IU/mL; Roche Diagnostic Systems, Branchburg, NJ, USA) before August 2008 and the COBAS AmpliPrep–COBAS TaqMan HBV test (lower limit of detection: 20 IU/mL) following that period. To maintain consistency in data interpretation, the VR was defined as a serum HBV DNA level of < 50 IU/mL.

### Detection of HBV-resistant mutants in HBV polymerase genes and genotypes

Mutations in the HBV DNA polymerase gene and HBV genotypes were assessed as described previously [31].

### Statistical analysis

Continuous variables are summarized as the median ± interquartile range; these variables were compared between the two groups through the Mann–Whitney U test. Categorical variables were analyzed with the chi-square or Fisher exact test, as applicable. Kaplan–Meier analysis and the log rank test were used to compare the cumulative risk of HCC among the patient subgroups. Multiple imputation was used to replace missing data on the analyzed variables to develop the prediction model [32]. The proposed HCC risk scoring system was developed using a risk scoring method [33]. A Cox proportional hazards model was used to estimate the multivariate-adjusted coefficients and hazard ratios for HCC risk predictors. Individual scores were computed by dividing the coefficient of each category for every risk predictor by that of every 10-year increase in age and then rounding to the nearest integer. Time-dependent ROC curves were used to calculate the AUROCs for evaluating the predictive accuracy of the HCC risk scores for each year. C-statistics were used to evaluate the overall performance of these scores. For the internal validation of the model, we used the bootstrap method to draw random samples, with the development group being replaced with 1000 replications to determine its C-statistics. The validation group was used for the external validation of the model. For calibrating the model, the Hosmer and Lemeshow goodness-of-fit test was used to assess the model fit between the observed and expected HCC rates in the patient subgroups. Statistical analyses were performed using SAS Version 9.4 (SAS Institute, Inc., Cary, NC, USA). The timeROC package provided in R Version 3.2.1 was used to plot the time-dependent ROC, calculate C-statistics, and compare the AUCs and C-statistics of two risk scores [34, 35]; a two-sided *P* < 0.05 was considered significant.

## SUPPLEMENTARY MATERIALS FIGURES AND TABLES





## Abbreviations

HBV: hepatitis B virus
HCC: hepatocellular carcinoma
ETV: entecavir
NA: nucleos(t)ide analog
CHB: chronic hepatitis B
VR: virological response
HBeAg: hepatitis B e antigen
AFP: alpha-fetoprotein
AUROC: area under the receiver operating characteristic curve
NPV: negative predictive value
CI: confidence interval
AST: aspartate aminotransferase
ALT: alanine aminotransferase
AAR: AST/ALT ratio
HBsAg: hepatitis B surface antigen.
