# Supplementary material for: Prediction model of hepatocellular carcinoma risk in Asian patients with chronic hepatitis B treated with entecavir
Source: Oncotarget. 2017 Sep 28;8(54):92431–41. doi: 10.18632/oncotarget.21369 (PMC5696194; doi:10.18632/oncotarget.21369)
Supplement: Supplementary file 1 [file oncotarget-08-92431-s001.pdf]

Prediction model of hepatocellular carcinoma risk in Asian patients with chronic hepatitis B treated with entecavir

SUPPLEMENTARY MATERIALS

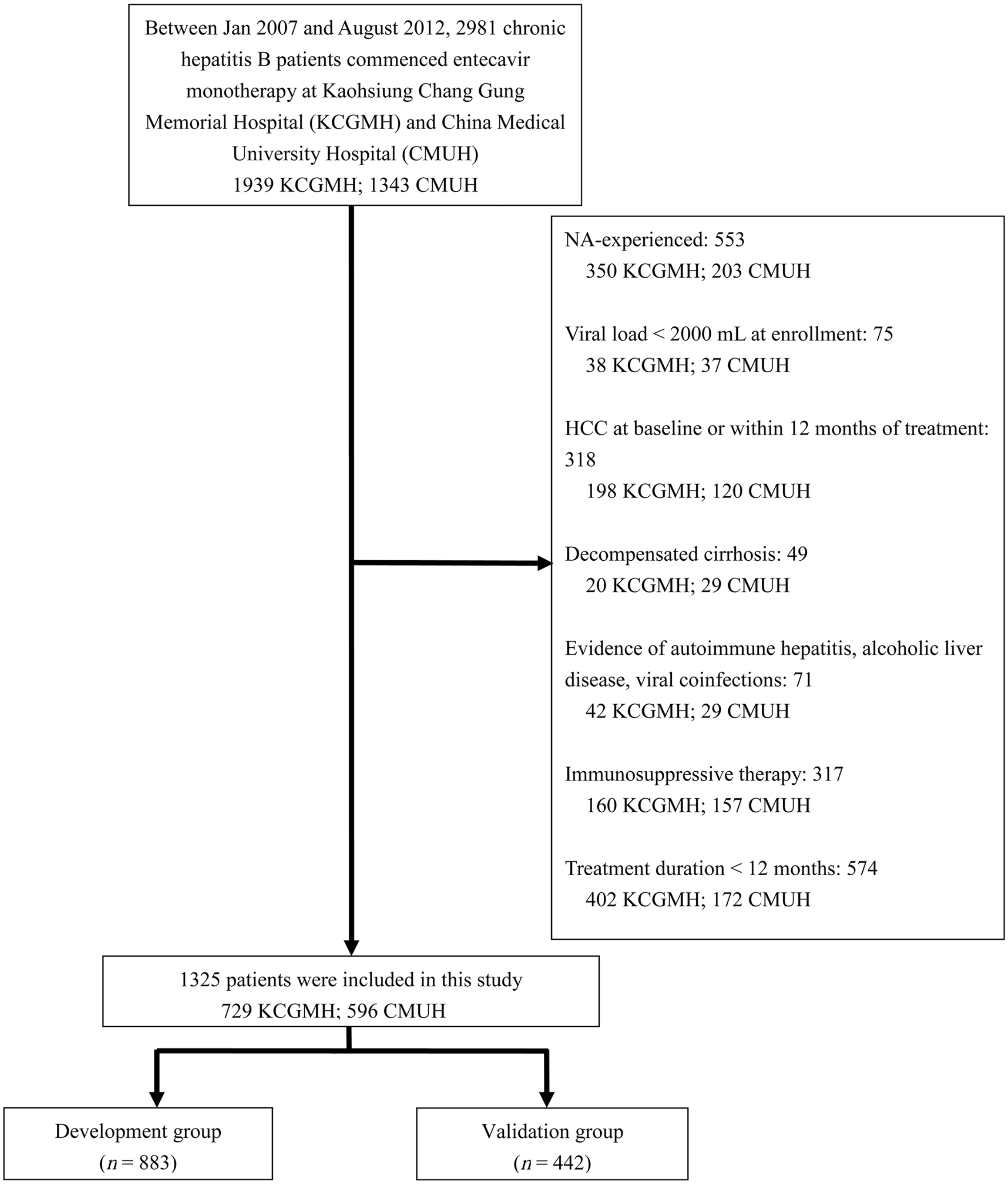

Supplementary Figure 1: Flow chart of patients enrolled in this study. HCC, hepatocellular carcinoma; NA, nucleos(t)ide analog.

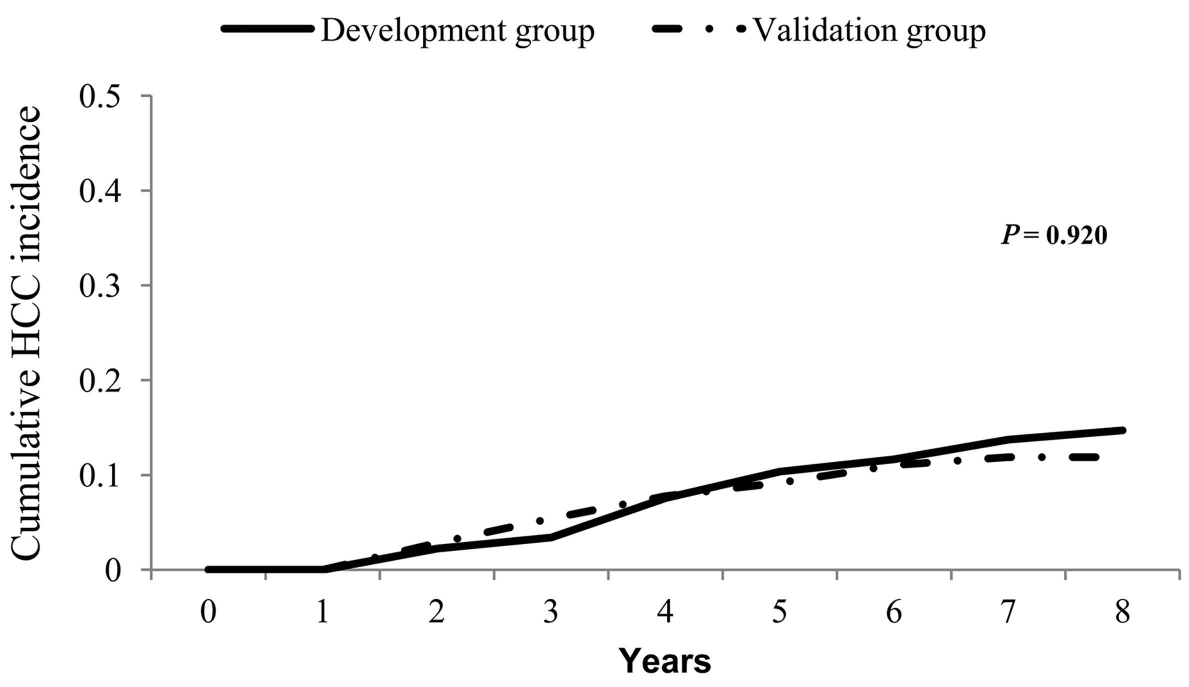

Number at risk

Development group	883	883	783	557	406	293	193	116	45
Validation group	442	442	399	272	212	166	108	60	23

Supplementary Figure 2: Cumulative incidence rates of HCC in the development and validation groups of CHB-naïve patients treated with entecavir. HCC, hepatocellular carcinoma; CHB, chronic hepatitis B.

Supplementary Table 1: Clinical characteristics of the development and validation groups.

See Supplementary File 1

Supplementary Table 2: Univariate and multivariate analyses of baseline factors associated with hepatocellular carcinoma in the development group (*n* = 883)

Variables	Univariate analysis		Multivariate analysis	
	Hazard ratio (95% CI)	*P* value	Hazard ratio (95% CI)	*P* value
Age (year)	1.058 (1.036–1.080)	< 0.001	1.048 (1.026–1.069)	< 0.0001
Sex, male *vs* female	1.015 (0.593–1.737)	0.958		
HBV genotype, C *vs* B	1.472 (0.919–2.357)	0.105		
HBeAg, yes or no	0.704 (0.412–1.205)	0.201		
HBV DNA, per log_{10} IU/mL	0.827 (0.704–0.971)	0.021		
Diabetes mellitus, yes *vs* no	2.640 (1.510–4.615)	0.001		
Family history of HCC, yes *vs* no	1.479 (0.846–2.586)	0.170		
Platelet, <90 *vs* ≥90 × 10^3/μL	3.636 (2.193–6.027)	< 0.001	2.716 (1.625–4.540)	0.0001
AST, per U/L	0.999 (0.997–1.000)	0.068		
ALT, per U/L	0.998 (0.996–0.999)	0.006		
AAR	2.590(1.825–3.677)	< 0.0001		
Total bilirubin, per mg/dL	0.947 (0.827–1.085)	0.435		
Albumin, per g/L	0.386 (0.260–0.573)	< 0.001		
INR, per ratio	2.088 (1.001–4.355)	0.050		
AFP at baseline, ≥5 *vs* <5 ng/mL	2.968 (1.625–5.423)	< 0.001	2.169 (1.171–4.018)	0.0139
HBsAg at baseline, per log_{10} IU/mL	0.793 (0.595–1.056)	0.112		

Abbreviations: AFP, alpha-fetoprotein; ALT, alanine aminotransferase; CI, confidence interval; AAR, AST/ALT ratio; HBV, hepatitis B virus; HBeAg, hepatitis B e antigen; HBsAg, hepatitis B surface antigen; HCC, hepatocellular carcinoma; INR, international normalized ratio; VR, virological response.

Supplementary Table 3: AUROC for predicting hepatocellular carcinoma within the initial 5 years of entecavir treatment by using different parameters in the development group (*n* = 883)

Factors	AUROC (95% CI)
Age at baseline	0.695 (0.631–0.759)*
Age at 12 months	0.695 (0.631–0.759)*
Platelet at baseline	0.773 (0.714–0.831)*†
Platelet at 12 months	0.832 (0.782–0.882)*†
AFP at baseline	0.365 (0.306–0.425)*‡
AFP at 12 months	0.776 (0.716–0.836)*‡
APA-B at baseline (logistic regression)	0.807 (0.755–0.859)*
APA-B at 12 months (logistic regression)	0.863 (0.819–0.908)*

*: $P < 0.05$ for the AFA-B score at 12 months *vs* any other parameter.
†: $P < 0.05$ for platelet at baseline *vs* platelet at 12 months of treatment.
‡: $P < 0.05$ for AFP at baseline *vs* AFP at 12 months of treatment.
Abbreviations: AFP, alpha-fetoprotein; AUROC, area under the receiver operating characteristic curve; CI, confidence interval.

Supplementary Table 4: Accuracy of predicting hepatocellular carcinoma within the initial 5 years of entecavir treatment in the development and validation groups by using the cutoff value of ≥6 in the APA-B score

	APA-B score ≥6	
	**Development (*n* = 883)**	**Validation (*n* = 442)**
Sensitivity	78.3%	90.3%
Specificity	80.0%	79.1%
Positive predictive value	22.2%	24.6%
Negative predictive value	98.1%	99.1%

Supplementary Table 5: *P* values of AUROC and C-statistic comparisons between the APA-B score and each risk score

	APA-B *vs* CU-HCC	APA-B *vs* REACH-B I	APA-B *vs* REACH-B II	APA-B *vs* PAGE-B
2 years	0.161	0.002	0.002	0.071
3 years	0.066	< 0.001	< 0.001	0.013
4 years	0.003	< 0.001	< 0.001	< 0.001
5 years	0.018	< 0.001	< 0.001	0.001
C-statistic	< 0.001	< 0.001	< 0.001	< 0.001

Abbreviations: AUROC, area under the receiver operating characteristic curve.
