# Supplementary material for: Prediction model of hepatocellular carcinoma risk in Asian patients with chronic hepatitis B treated with entecavir
Source: Oncotarget. 2017 Sep 28;8(54):92431–41. doi: 10.18632/oncotarget.21369 (PMC5696194; doi:10.18632/oncotarget.21369)
Supplement: Supplementary file 2 [file oncotarget-08-92431-s002.pdf]

Table 1: Clinical characteristics of the development and validation groups

Variable<br>Median ± IQR or *n* (%)	Development<br>(*n* = 883)	Validation<br>(*n* = 442)	*P* value
Age (year)	50 ± 17	49 ± 18	0.483
Sex			0.377
Female	248 (28.1)	114 (25.8)	
Male	635 (71.9)	328 (74.2)	
HBeAg			0.500
Negative	572 (64.8)	278 (62.9)	
Positive	311 (35.2)	164 (37.1)	
Cirrhosis status			0.668
No cirrhosis	566 (64.1)	278 (62.9)	
Cirrhosis	317 (35.9)	164 (37.1)	
Diabetes mellitus			0.258
No	784 (88.8)	383 (86.7)	
Yes	99 (11.2)	59 (13.3)	
Family history of HCC			0.340
No	751 (85.1)	367 (83)	
Yes	132 (14.9)	75 (17)	
Albumin, g/dL	4.11 ± 0.6	4.1 ± 0.6	0.284
AST, U/L	72 ± 117	72 ± 105	0.564
ALT, U/L	106 ± 215	107 ± 205	0.670
AAR	0.7 ± 0.4	0.7 ± 0.4	0.450
Total bilirubin, mg/dL	1 ± 0.78	1 ± 0.8	0.790
INR	1.07 ± 0.14	1.09 ± 0.16	0.122
Platelet, × 10^3/μL	163 ± 79	162 ± 74	0.793
Platelet at 12 months, × 10^3/μL	163 ± 81	165 ± 84	0.676
AFP at baseline, ng/mL	6.17 ± 10.46	5.57 ± 10.06	0.377
AFP at 12 months, ng/mL	3.38 ± 2.36	3.45 ± 2.31	0.837
Genotype			0.947
B	573 (64.9)	286 (64.7)	
C	310 (35.1)	156 (35.3)	
HBV DNA, $\log_{10}$ IU/mL	5.95 ± 2.3	6.02 ± 2.45	0.479

HBsAg at baseline, log_{10} IU/mL	3.29 ± 0.96	3.3 ± 0.85	0.993
HBsAg at 12 months, log_{10} IU/mL	3.09 ± 0.72	3.08 ± 0.7	0.769
VR during treatment			0.997
No	24 (2.7)	12 (2.7)	
Yes	859 (97.3)	430 (97.3)	
Time to VR (month)	6.0 ± 6.1	6.0 ± 6.5	0.697

Abbreviations: AFP, alpha-fetoprotein; ALT, alanine aminotransferase; AST, aspartate aminotransferase AAR, AST/ALT ratio; HBV, hepatitis B virus; HCC, hepatocellular carcinoma; HBeAg, hepatitis B e antigen; HBsAg, hepatitis B surface antigen; INR, international normalized ratio; IQR, interquartile range; VR, virological response.
